# Oral Nonviral Gene Delivery for Chronic Protein Replacement Therapy

**DOI:** 10.1002/advs.201701079

**Published:** 2018-06-06

**Authors:** Po‐Yen Lin, Ya‐Ling Chiu, Jing‐Huei Huang, Er‐Yuan Chuang, Fwu‐Long Mi, Kun‐Ju Lin, Jyuhn‐Huarng Juang, Hsing‐Wen Sung, Kam W. Leong

**Affiliations:** ^1^ Department of Chemical Engineering/Institute of Biomedical Engineering National Tsing Hua University Hsinchu 30013 Taiwan (ROC); ^2^ Department of Biomedical Engineering/Department of Systems Biology Columbia University New York NY 10027 USA; ^3^ Graduate Institute of Biomedical Materials and Tissue Engineering Taipei Medical University Taipei 11031 Taiwan (ROC); ^4^ Department of Biochemistry and Molecular Cell Biology School of Medicine College of Medicine Taipei Medical University Taipei 11031 Taiwan (ROC); ^5^ Department of Nuclear Medicine and Molecular Imaging Center Chang Gung University and Memorial Hospital Taoyuan 33305 Taiwan (ROC); ^6^ Division of Endocrinology and Metabolism Chang Gung University and Memorial Hospital Taoyuan 33305 Taiwan (ROC)

**Keywords:** chronic protein replacement therapy, diabetes mellitus, gene therapy, nonviral vectors, oral delivery

## Abstract

Efficient nonviral oral gene delivery offers an attractive modality for chronic protein replacement therapy. Herein, the oral delivery of insulin gene is reported by a nonviral vector comprising a copolymer with a high degree of substitution of branched polyethylenimine on chitosan (CS‐g‐bPEI). Protecting the plasmid from gastric acidic degradation and facilitating transport across the gut epithelium, the CS‐g‐bPEI/insulin plasmid DNA nanoparticles (NPs) can achieve systemic transgene expression for days. A single dose of orally administered NPs (600 µg plasmid insulin (pINS)) to diabetic mice can protect the animals from hyperglycemia for more than 10 d. Three repeated administrations spaced over a 10 d interval produce similar glucose‐lowering results with no hepatotoxicity detected. Positron‐emission‐tomography and computed‐tomography images also confirm the glucose utilization by muscle cells. While this work suggests the feasibility of basal therapy for diabetes mellitus, its significance lies in the demonstration of a nonviral oral gene delivery system that can impact chronic protein replacement therapy and DNA vaccination.

## 1 Introduction

Oral gene delivery may offer many interesting therapeutic options. Genes may be orally delivered to treat local disorders such as inflammatory bowel disease^1^ and colon cancer,^2^ as well as systemic diseases such as hemophilia.^3^ In addition, it would be an interesting modality for DNA vaccination because of an abundance of immune inductive tissues in the gastrointestinal (GI) tract.^2^ It is also the most attractive route of delivery because of patient compliance and repeatable administration. To translate, nonviral delivery would be the way to go. However, despite glimpses of promise,^4^, ^5^ success of nonviral oral gene delivery has been elusive.

Oral delivery of gene vectors faces one of the most challenging hurdles because of the physiological (harsh gastric pH and many degrading enzymes) and anatomical (mucosal epithelium) barriers in the GI tract. Furthermore, the rapid self‐renewal of the intestinal epithelial cells (2–3 d) would prevent sustained therapeutic gene expression.^6^ Therefore, to treat chronic diseases such as diabetes mellitus, nonviral vectors must be delivered across the mucosal epithelium, transported through the bloodstream, and then accumulated in the systemic tissues to have a chance of prolonging the typical transgene expression observed for several days.

Chitosan (CS) is an attractive gene carrier because of its low toxicity to cells^7^ and tunable physicochemical characteristics. Varying the molecular weight (*M*_W_) and degree of deacetylation (DA) of CS can yield a polysaccharide with different biodegradability and charge density at physiological pH. Cationic at pH below 6, CS can readily complex plasmid DNA (pDNA), small interfering RNA (siRNA) or micro RNA (miRNA) to form nanoparticles (NPs; polyplex). However, the in vitro transfection efficiency of CS is mediocre compared with many other nonviral gene carriers. Slow unpacking of the CS polyplex intracellularly is one of the main culprits.^8^, ^9^, ^10^ Yet, despite more than two decades of research for an alternative, there has been no better candidate than CS in oral gene delivery. Chemical stability and hence slow unpacking of the polyplex that contributes to poor in vitro transfection might have helped navigation through the oral route with minimal degradation. The known mucoadhesiveness of CS might also have contributed to longer retention in the GI tract to facilitate uptake and transport across the gut epithelium.^11^ This has led to the use of CS for the development of oral DNA vaccines. However, the transfection efficiency of CS must be improved for any chance of translation. Polyethylenimine (PEI) is one of the most potent gene carriers owing to its high buffering capacity at the acidic endosomal pH.^12^ The standard PEI composition of a branched structure (bPEI) with a high *M*_W_ (25 kDa) is nevertheless too toxic for in vivo application.^13^ bPEI of lower *M*_W_ (600–1800 Da) is less toxic but is also less efficient.^14^

**Figure 1:**
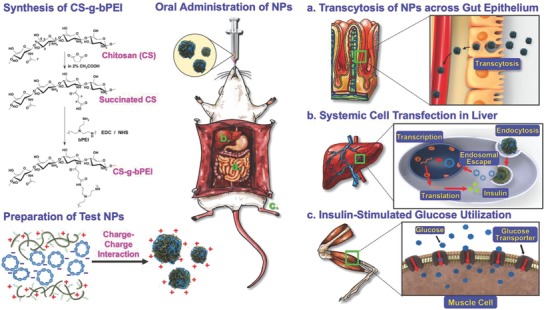
Synthesis of CS‐g‐bPEI copolymer, preparation of CS‐g‐bPEI/pDNA NPs, oral administration of as‐prepared NPs using an oral feeding needle, transcytosis of NPs across gut epithelium, systemic cell transfection in liver to express insulin, and insulin‐stimulated glucose utilization in muscle cells in diabetic mice.

Although previous studies have investigated the grafting of bPEI to CS to improve the transfection efficiency of CS, it was only after extensive screening that we came up with a composition comprising high‐density PEI grafting to low‐*M*_W_ CS that could achieve effective oral nonviral gene delivery. In this study, we report the development of a nonviral oral gene carrier comprising CS (*M*_W_ = 15 kDa and DA = 85%) grafted with bPEI (*M*_W_ = 0.8 kDa) at a high grafting degree of substitution (DS ≈ 40%) (CS‐g‐bPEI) for the oral delivery of insulin pDNA. After in vitro characterization and in vivo optimization with the green fluorescent protein (GFP) reporter gene, we show that CS‐g‐bPEI/pDNA NPs can effectively deliver a human insulin plasmid to reduce blood glucose levels in mice with streptozotocin (STZ)‐induced diabetes (**Figure** **1**). A single dose of 600 µg of insulin pDNA in NPs delivered by oral gavage can lower the blood glucose in diabetic mice to 50–80% of the baseline level for 10 d. Two more repeated dosing spaced 10 d apart can produce the same therapeutic effect in lowering the glucose level without detectable hepatotoxicity.

## 2 Results

### 2.1 Synthesis and Characterization of CS‐g‐bPEI

**Figure 2:**
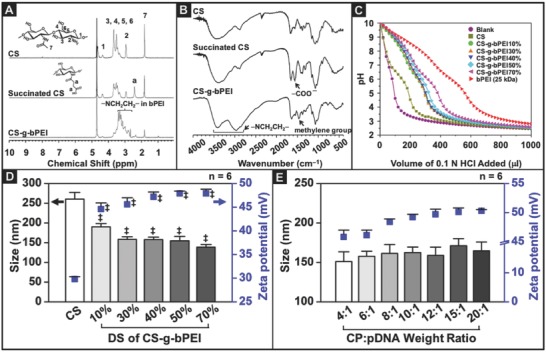
Physiochemical properties of CS‐g‐bPEI copolymer and CS‐g‐bPEI/pDNA NPs. Characteristics of CS, succinated CS, and CS‐g‐bPEI: A) ^1^H NMR spectra and B) Fourier‐transform infrared spectroscopy(FT‐IR) spectra. C) Buffering capacities of CS, bPEI (25 kDa), and CS‐g‐bPEI with various DSs of bPEI. Size and zeta potential of CS‐g‐bPEI/pDNA NPs that were prepared using D) CS‐g‐bPEI:pDNA weight ratio fixed at 6:1 with various DS of bPEI and E) different CS‐g‐bPEI40%:pDNA weight ratios (*n* = 6 in each group; error bars represent standard deviation). ‡: Statistically significant versus CS group (*P* < 0.05).

Conventional methods of grafting PEI to CS are based on heterogeneous reactions, typically resulting in a low‐grafting DS (≈5–20%).^15^, ^16^ In this study, the CS‐g‐bPEI copolymer was synthesized using a two‐step process in a homogeneous aqueous environment. Briefly, the CS (15 kDa) was first reacted with predetermined amounts of succinic anhydride to yield succinated CS that had a distinct DS of succinyl groups at the *N*‐positions on its backbone (Figure 1). The low‐*M*_W_ bPEI (0.8 kDa) was then conjugated to the carboxyl group of the as‐prepared succinated CS in the presence of excess 1‐ethyl‐3‐(3‐dimethylaminopropyl) carbodiimide (EDC)/*N*‐hydroxysuccinimide (NHS), ensuring that almost all the succinyl groups were coupled with bPEI (CS‐g‐bPEI). The succinated CS spectrum yielded a signal at 2.4 ppm that was absent from the ^1^H NMR spectrum of CS and could be attributed to the methylene protons on succinyl groups, verifying the substitution of the succinyl group onto the CS backbone (**Figure** **2**A). The DS of succinyl groups in the CS structure was obtained from the ratio of the integral peak of the methylene protons (—CH_2_CH_2_—) on succinyl groups at 2.4 ppm and the integral peak of H‐2 proton on the CS backbone at 3.0 ppm. Using a homogeneous instead of a heterogeneous reaction condition to obtain higher yield and better control of the DS,^17^ a series of succinated CS with various DS (≈10%, 30%, 40%, 50%, and 70%) was synthesized and showed strong proton signals from 2.67 to 3.26 ppm, which were assigned to the broad peak (—NCH_2_CH_2_—) of bPEI.^18^

The results of FT‐IR spectroscopy were consistent with those obtained from ^1^H NMR analysis (Figure 2B). The FT‐IR spectrum of the succinated CS exhibited a peak at 1567 cm^−1^ that was absent from the CS spectrum, corresponding to the carboxyl group (—COO^−^) in the succinyl group (Figure 2B). In the spectrum of CS‐g‐bPEI, the absorption peak at 1567 cm^−1^ was significantly diminished, while the characteristic peaks of bPEI at 1470 cm^−1^ (methylene C—H bending), 2979 cm^−1^ (methylene N—H bending), and 3415 cm^−1^ (N—H stretch)^16^ were obtained, confirming that the succinyl groups on the succinated CS were coupled with bPEI.

### 2.2 Buffering Capacity of CS‐g‐bPEI

An acid–base titration assay was used to determine the buffering capacities of unmodified CS, bPEI (25 kDa), and the CS‐g‐bPEI copolymers with different DS over the pH range of 10–2.5. As shown in Figure 2C, CS exhibited poor buffering capacity, which might explain its low transfection efficiency. Grafting bPEI (0.8 kDa) to CS increased the buffering capacity in a DS‐dependent manner, although not quite matching the superior buffering capacity of the 25 kDa bPEI.

### 2.3 Particle Size and Zeta Potential of CS‐g‐bPEI/pDNA NP

The particle size and zeta potential of CS‐g‐bPEI/pDNA NPs (formulated with plasmid enhanced green fluorescent protein (pEGFP)) were characterized by dynamic light scattering (DLS). As shown in Figure 2D, with the copolymer:pDNA (CP:pDNA) weight ratio fixed at 6:1, CS‐g‐bPEI showed smaller sizes compared with CS; even at as low as 10% DS, the size was reduced by 20%, reflecting a more compact structure enabled by the higher cationic charge density. Further reduction was small but detectable as the DS exceeded 50% (*P* < 0.05). The zeta potential increased with DS in an expected manner correspondingly. With the DS fixed at 40%, size and zeta potential increased with the CP:pDNA weight ratio but only slightly (Figure 2E).

### 2.4 Transfection Efficiency and Cytotoxicity of CS‐g‐bPEI/pDNA NPs

**Figure 3:**
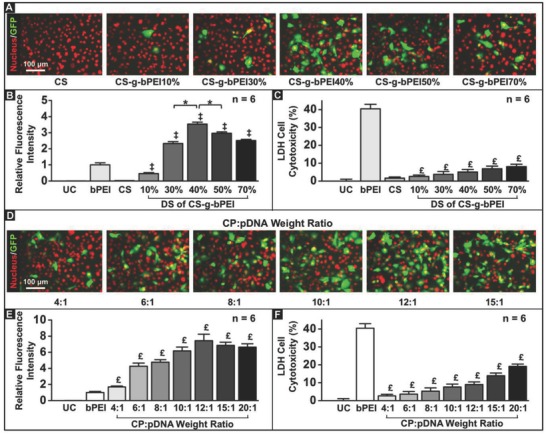
Optimization of formulations of CS‐g‐bPEI/pDNA NPs with respect to transfection efficiency and cytotoxicity. Effects of DS of bPEI on CS‐g‐bPEI: A) fluorescent images; B) transfection efficiency; and C) LDH cell cytotoxicity. Effects of CS‐g‐bPEI40%:pDNA weight ratio: D) fluorescent images; E) transfection efficiency; and F) LDH cell cytotoxicity. *: Statistically significant (*P* < 0.05); ‡: Statistically significant versus CS group (P < 0.05). £: Statistically significant versus bPEI group (*P* < 0.05).

Evaluated against HT1080 cells and in a serum‐enriched medium (10% fetal bovine serum; FBS), which is commonly used to evaluate the serum resistance of nonviral vectors before they are used in vivo.^19^ Fluorescence‐activated cell sorter analysis showed the best performance with CS‐g‐bPEI‐40% DS at the CP:pDNA ratio of 6:1 (**Figure** **3**A,B). Notably it outperformed the 25 kDa bPEI with a much lower cytotoxicity (Figure 3C). Keeping the DS constant at 40%, the transfection efficiency also increased with the CP:pDNA ratio, peaking at around 10:1 to 15:1 and again outperforming 25 kDa bPEI with as much as 7.5‐fold increase in fluorescence intensity (Figure 3D,E). The cytotoxicity did rise with the CP:pDNA ratio but still more than twofold lower than 25 kDa bPEI even at the highest ratio of 20:1. The above results demonstrate that the test NPs with a CP:pDNA weight ratio of 12:1 had the highest level of gene expression (Figure 3E) and limited lactate dehydrogenase (LDH) cell toxicity (Figure 3F) and were therefore chosen for subsequent experiments.

### 2.5 Physical Stability of CS‐g‐bPEI/pDNA NPs in Environments with Particular pH

**Figure 4:**
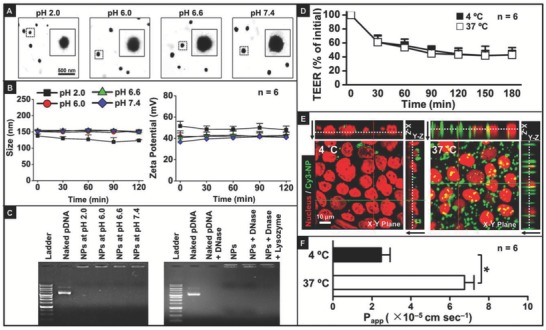
Physical stability, pDNA protection, and transcytosis capability of CS‐g‐bPEI/pDNA NPs in distinct in vitro environments that simulate their transport through GI tract. Physical stability of NPs at various pH conditions: A) TEM images and B) particle size and zeta potential; C) pDNA protection of NPs in various pH environments and nuclease digestion using DNase I and lysozyme. Transcytosis capability of NPs in Caco‐2 cell monolayers at 4 or 37 °C, evaluated by D) reduction of TEER, E) transcellular penetration, visualized using constructed 3D CLSM images, and F) *P*_app_ values. Black arrows indicate direction from apical layer to basolateral layer. *: Statistically significant (*P* < 0.05).

The physical stability of CS‐g‐bPEI/pDNA NPs in the pH range of 2.0–7.4, representing the pH conditions in various segments of the GI tract, was characterized by transmission electron microscopy (TEM) and DLS. **Figure** **4**A,B shows that the CS‐g‐bPEI/pDNA NPs maintained their shape under all examined pH conditions; additionally, the DLS data revealed that the particle size and zeta potential remained approximately constant across the pH range.

### 2.6 Protection of pDNA by CS‐g‐bPEI against Acidic pH and Enzymatic Degradation

A gel retardation assay was conducted to evaluate the protection of pDNA by the CS‐g‐bPEI copolymer against the gastric pH environment and the intestinal enzymes. In this study, the CS‐g‐bPEI/pDNA NPs were maintained under various GI pH conditions or subjected to nuclease degradation using DNase I as a model enzyme; the degradability of the NPs that were prepared with CS‐g‐bPEI against lysozyme, an intestinal enzyme, was also explored.^20^ According to Figure 4C, at all tested pH values, the NPs retained all of the pDNA in the agarose gel slots, suggesting that the CS‐g‐bPEI copolymer effectively protected its condensed pDNA even under extreme gastric pH conditions. Following enzymatic degradation, DNase I degraded all naked pDNA. In contrast, the pDNA entrapped by the NPs, even after the same treatment with DNase I, remained intact at the top of the lanes, indicating that the cationic CS‐g‐bPEI copolymer bound and protected the pDNA from degradation efficiently.^21^

### 2.7 Transcytosis of CS‐g‐bPEI/pDNA NPs

The ability of CS‐g‐bPEI/pDNA NPs to undergo transcytosis across the intestinal epithelium was evaluated using the Caco‐2 cell Transwell model.^22^ Transcytosis is an energy‐dependent transport process, which is extremely sensitive to the environmental temperature.^23^ CS‐g‐bPEI/pDNA NPs were added to the donor compartment of Caco‐2 cell monolayers at the physiological temperature (37 °C) or a low temperature (4 °C). The transepithelial electrical resistance (TEER), which reflects the ion permeability of the monolayers,^22^ was monitored using a voltmeter with a chopstick electrode. Figure 4D plots the TEER data expressed as percentage of the initial value at each time point. It shows the progressive decrease of TEER at either test temperature as the positively charged NPs bound to the Caco‐2 monolayer and increased the paracellular permeability^24^ to reduce the TEER.

Confocal laser scanning microscopy (CLSM) was used to elucidate the transport mechanism of CS‐g‐bPEI NPs across the Caco‐2 cells in monolayers; in this study, pDNA was fluorescently labeled with Cy3. Figure 4E presents orthogonal CLSM images of Caco‐2 cell monolayers that had been exposed to the fluorescence‐labeled NPs (green) for 3 h. As indicated in the *X*–*Z* and *Y*–*Z* vertical CLSM images, green fluorescence signals infiltrated the apical layer of the cells (endocytosis of the transported NPs), passed across the cells, and through their basolateral layer (exocytosis of the NPs). Notably, the intensity of the fluorescence signals in cell monolayers that had been incubated at 37 °C was much stronger than that incubated at 4 °C, revealing that reducing the temperature markedly reduced the rate of transcytosis of the test NPs, reflective of an energy‐dependent transport process. After 3 h of incubation, the apparent permeability coefficient (*P*_app_), which is defined as the rate of the appearance of the test NPs in the receiving compartment,^22^ was measured. Figure 4F shows that the *P*_app_ value dropped from 6.76 × 10^−5^ to 2.48 × 10^−5^ cm s^−1^ (*P* < 0.05) as the temperature was decreased from 37 to 4 °C, corroborating with the CLSM observation that the transcytosis of CS‐g‐bPEI/pDNA NPs proceeds by an energy‐dependent mechanism.

### 2.8 Systemic Accumulation of CS‐g‐bPEI/pDNA NPs and Their Transgene Expression

**Figure 5:**
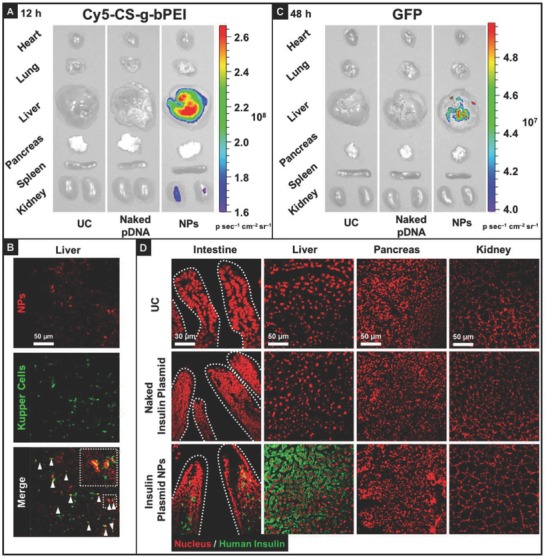
Biodistribution and tissue expression kinetics of CS‐g‐bPEI/pDNA NPs in mice: A) ex vivo fluorescence images of accumulation of test NPs in isolated major organs 12 h following oral administration of naked pDNA or NPs; B) colocalization of test NPs and Kupffer cells in hepatic sections; C) ex vivo fluorescence images of expression of test NPs in isolated major organs 48 h after oral administration of naked pDNA or NPs; D) immunostaining of insulin in major tissues following oral administration of a single dose of naked insulin plasmid or insulin plasmid NPs.

Following the oral treatment of mice with suspensions of CS‐g‐bPEI/pDNA NPs in deionized (DI) water, the biodistribution of the NPs was examined using an in vivo imaging system (IVIS). To trace the NPs and evaluate their transgene expression in systemic tissues, the CS‐g‐bPEI copolymer was fluorescently labeled with Cy5, and pEGFP was used as a model gene. Twelve hours after oral treatment (**Figure** **5**A), Cy5‐CS‐g‐bPEI/pEGFP NPs were detected in systemic tissues, including the liver and kidney, indicating that at least a fraction of the orally administered NPs crossed the intestinal barrier and entered systemic circulation. Immunofluorescent analysis using the macrophage marker F4/80 indicated that the CS‐g‐bPEI/pDNA NPs were internalized by F4/80^+^ Kupffer cells (as indicated by the white arrowheads in Figure 5B) or remained along hepatic sinusoids. Forty‐eight hours after oral treatment, GFP expression was clearly observed in the liver (Figure 5C). Although one group reported that the kidney would collect more systemically circulating particles than other organs as it receives a large amount of blood from the heart,^25^ we did not detect any gene expression in the kidney, consistent with other reports that probably the kidney is perfused by capillaries with a barrier of continuous endothelia.^26^, ^27^

### 2.9 Therapeutic Effects in Diabetic Mice

To explore the potential of the above CS‐g‐bPEI NP formulation for translational applications, the formulation was further used in the oral delivery of a human insulin plasmid (INSL4) to reduce the blood glucose levels in mice with STZ‐induced diabetes. Based on Figure 5D, no gene expression was observed in any tissues of the group that was treated with naked insulin plasmid. In contrast, in the group that received the insulin plasmid NPs, immunofluorescence staining revealed the expression of human insulin in local tissues (intestinal villi) as well as systemic tissues (liver), indicating that the effects of this NP formulation for oral delivery are not merely local.

### 2.10 Lowering of Blood Glucose Levels by CS‐g‐bPEI/pDNA NPs

**Figure 6:**
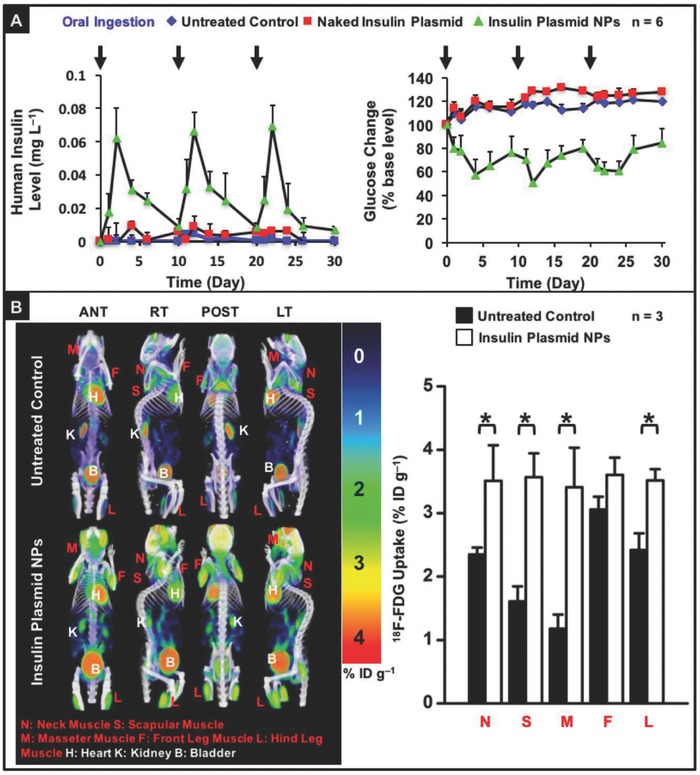
Human insulin expression and insulin‐stimulated glucose utilization in diabetic mice that had been orally treated with DI water (untreated control), naked insulin plasmid, or insulin plasmid NPs: A) expressed plasma human insulin level and variation in blood glucose level vs. time; B) PET/CT images that show ^18^F‐FDG uptake and semiquantitative accumulated radioactivity concentrations in muscle tissues. Images display anterior (ANT), right lateral (RT), posterior (POST), and left lateral (LT) views, in terms of standardized uptake value (SUV). *: Statistically significant (*P* < 0.05).

A study was conducted to determine whether the level of insulin that was expressed by the CS‐g‐bPEI/pDNA NPs could lower the blood glucose levels in diabetic mice. According to **Figure** **6**A, a single dose of orally administered NPs was capable of ensuring the expression of a significant level of human insulin in plasma for about 10 d, effectively lowering the blood glucose level. Notably, similar results could also be obtained by repeatedly dosing the diabetic mice with the NPs once every 10 d.

### 2.11 Enhancement of ^18^F‐Fluorodeoxyglucose Utilization in Muscle Cells

Insulin can stimulate the utilization of glucose in muscle cells by promoting the translocation of the glucose transporter 4 (Glut4) from its intracellular site to the plasma membrane.^28^ To examine the insulin‐stimulated glucose utilization in diabetic mice, 2 d following the oral administration of insulin plasmid NPs, ^18^F‐fluorodeoxyglucose (^18^F‐FDG), which has been clinically used as a radiopharmaceutical analog of glucose,^28^ was administered intravenously; mice that received no test NPs served as a control. The biodistribution of the insulin‐stimulated ^18^F‐FDG utilization was investigated by animal positron‐emission‐tomography (PET) and computed‐tomography (CT) scanners.

Figure 6B and Movie S1 (Supporting Information) show the 3D maximum‐intensity projection images reconstructed from animal PET/CT images with anterior, right lateral, posterior, and left lateral views. A higher ^18^F‐FDG uptake, indicating a higher glucose utilization, was observed in the neck muscle, scapular muscle, masseter muscle, front leg muscle, and hind leg muscle in the group that was treated with the insulin plasmid NPs than in the group that received no NPs (untreated control, *P* < 0.05).

### 2.12 Acute Liver Toxicity

**Figure 7:**
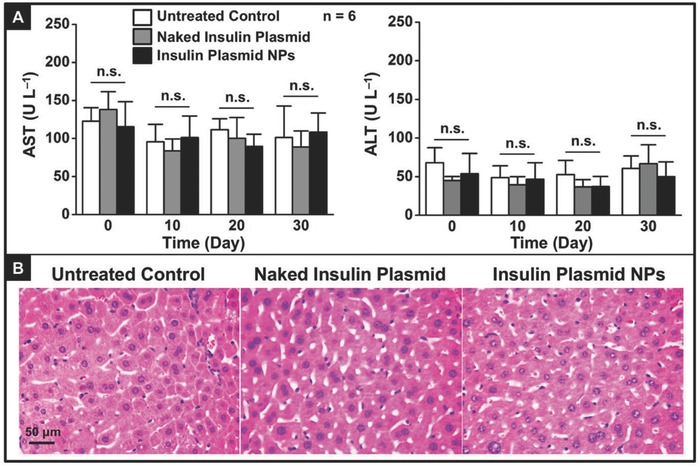
Effects of repeated oral administration of DI water, naked insulin plasmid, or insulin plasmid NPs on liver function and histology in diabetic mice: A) serum ALT and AST levels versus time and B) representative photomicrographs of liver section (H&E staining). n.s.: Not significant (*P* > 0.05).

To determine whether the CS‐g‐bPEI/pDNA NPs accumulated in the liver would induce any acute liver toxicity, blood samples were taken and analyzed to evaluate serum alanine aminotransferase (ALT) and aspartate aminotransferase (AST) activities at various time points prior to the repeated oral administration of the test NPs, as well as at the end of the study. The mice that received naked insulin plasmid and the untreated mice were controls. As displayed in **Figure** **7**A, both the ALT and the AST levels were similar across all studied groups (*P* > 0.05). Histological assessments of the harvested liver tissue samples were carried out to determine whether the test NPs caused tissue damage or inflammation. According to Figure 7B, hepatocytes in the liver samples appeared normal, and no significant inflammatory infiltrates were observed.

## 3 Discussion

Gene therapy is enjoying a resurgence since the approval of using adeno‐associated virus to treat the rare genetic disease of lipoprotein lipase deficiency by the European Medicines Agency in 2012. A number of late‐stage clinical trials using viral vectors to treat diseases including retinal dystrophy, spinal muscular atrophy, and to X‐linked adrenoleukodystrophy are also in the pipeline.^29^ While this exciting development is realizing the potential of gene therapy, particularly for one‐time administration, chronic applications may still require nonviral gene vectors to deliver. However, nonviral gene delivery faces the intrinsic weakness of low transfection efficiency in vivo coupled with transient transgene expression. It would require frequent administrations to be effective. This would, in turn, dictate that the administration route be convenient to render nonviral gene therapy for chronic applications attractive. Oral administration would be the top choice.

The oral administration of nonviral vectors for gene delivery must overcome various difficulties in their transport through the GI tract, such as the physical stability of the vectors under particular pH conditions, the protection of the carried pDNA in harsh GI environments, and the transcytosis of the vectors through the intestinal epithelium. Since the p*K*_a_ of bPEI is around 8.4,^30^ the CS‐g‐bPEI copolymer exhibits positive charges over a wide range of pH values, rendering the complex of CS‐g‐bPEI and pDNA stable in the various pH milieus in the GI tract (Figure 4A,B).

Naked nucleic acids can be denatured at the gastric acidic pH and degraded by enzymes in the intestinal tract, reducing their effectiveness; these effects potentially inhibit functional delivery to the target cells.^31^ The gel retardation assay indicated that the cationic CS‐g‐bPEI copolymer had strong pDNA binding ability and was highly protective of pDNA; therefore, the CS‐g‐bPEI carrier effectively protected pDNA from acidic denaturation and enzymatic degradation in the harsh environment of the GI tract. Increased particle stability against gastric pH and intestinal enzyme degradation during transport would enhance the probability of uptake into target cells, increasing transfection efficiency.

A prerequisite for effective systemic cell transfection in vivo is that the orally administered vectors must be transported across the mucosal epithelium and enter the bloodstream, eventually accumulating in systemic tissues. A potential mechanism of the transport of CS‐g‐bPEI NPs through the intestinal epithelium was investigated in vitro in Caco‐2 cell monolayers. Based on Figure 4D,F, an energy‐dependent mechanism of the transport of CS‐g‐bPEI/pDNA NPs across Caco‐2 cells was identified, and the transport mechanism involved transcytosis. This is consistent with the literature that NPs with a positive zeta potential are generally effective in transcytosis.^32^

Ex vivo fluorescence images reveal accumulation of the orally administered Cy5‐CS‐g‐bPEI/pEGFP NPs in the liver (Figure 5A), verifying that some of the test particles had been absorbed into the bloodstream via the epithelial cells along the intestinal villi, from where they accessed the liver. Upon intestinal absorption, nutritional compounds are transported directly to the liver via the hepatic portal vein.^33^ The mammalian liver contains many resident macrophages (Kupffer cells), which are efficient in the uptake and retention of foreign particles that circulate in the bloodstream (Figure 5B).^34^ Transgene expression was clearly observed subsequently in the liver (Figure 5C), suggesting that Kupffer cells were one of the cell types that expressed the insulin. The epithelial cells along the intestinal villi may also have functioned as protein factories to secrete the insulin into the bloodstream (Figure 5D).

Insulin expressed in the liver is essential for regulating glucose homeostasis.^33^ Physiologically, the insulin that is endogenously secreted by the pancreatic islets of Langerhans is transported to the liver by portal circulation.^28^ The in vivo studies demonstrated that the orally administered insulin plasmid NPs resulted in sustained therapeutic gene expression in mice with STZ‐induced diabetes, and the level of insulin expression was sufficient to ameliorate hyperglycemia in the diabetic mice over 10 d (Figure 6A). We showed that the expressed insulin increased the concentration of glucose transporters (Glut4) at the plasma membrane of the muscle cells,^28^ facilitating their glucose (^18^F‐FDG) utilization (Figure 6B), and thereby lowering blood glucose levels. One advantage of gene therapy is that it can reduce the number of administrations required to maintain a therapeutic drug concentration.^35^ Sufficient expression of insulin in the diabetic mice was further verified following repeated oral delivery of the insulin plasmid NPs, suggesting that this new nonviral gene delivery system may be applicable for chronic protein replacement therapy.

In the safety study, repeated three‐time dosing with insulin plasmid NPs did not induce any significant variation in serum ALT/AST activities or any detectable histological changes in the liver (Figure 7A,B), indicating that there was no issue with hepatotoxicity of these NPs.

One limitation of nonviral oral gene delivery is the lag time between the feeding and the transgene expression. Although this is no different from oral medication versus intravenous injection in drug therapy, the delay is exacerbated in gene therapy because of all the intracellular barriers and molecular processing in the gene transfer process. Therefore, it would not be able to meet the requirements of on‐demand and quick response in diabetes therapy; it would be suitable only for a basal supplement to reduce the injections required and prevent hypoglycemic accidents. However, this issue would not be serious in chronic protein replacement therapy since frequent oral dosing is possible. Another limitation of this study is that we have not explored macroformulation to improve the delivery of the NPs to the GI tract because oral formulation technologies are difficult to apply in mice. In larger animals, and of course in humans, the NPs can be packed in gelatin capsules with enteric coating and excipients to protect the NPs from degradation in transit through the stomach. Macroformulation will likely further improve the efficacy of this oral gene delivery system.

It augers well that the CS‐g‐bPEI copolymer developed in the study may be applicable for the repeated oral administration of other therapeutic genes. Building on this low‐toxicity finding, it is tempting to speculate that this new gene delivery system may find applications in siRNA or miRNA therapy. However, it remains to be seen if the high PEI grafting density, which functions well in this study but may bind the shorter siRNA or miRNA more tightly, would produce the favorable intracellular unpacking kinetics to generate therapeutic effects. Nevertheless, the grafting density of PEI on CS can be readily optimized with the chemistry shown in this study.

The translational potential of this nonviral oral gene delivery system will need to be evaluated in further preclinical experiments involving large animals and long‐term studies to demonstrate its safety and efficacy. Additionally, the biomanufacturing issue which involves the large‐scale production of these polymeric NPs and quality control must also be addressed. Recent studies suggest that flash nanocomplexation may be particularly suitable for the manufacturing of these NPs.^36^, ^37^ It has been 20 years since the first report suggesting the feasibility of using nonviral vectors to generate a therapeutic effect through oral administration.^7^ The ensuing years have only produced promise plagued by modest efficacy and low reproducibility. This study shows that a high charge density on CS is crucial for overcoming these drawbacks.

## 4 Conclusions

Although CS has been studied extensively as a gene carrier in the past two decades, it has been plagued by poor efficacy and reproducibility in oral gene delivery until the current study showing that a high grafting density of low‐molecular‐weight bPEI on CS can overcome the challenging oral delivery barriers. In this study, we showed efficacy in protecting diabetic mice from hyperglycemia for more than 10 d with a single dose of NPs (600 µg plasmid insulin (pINS)) delivered orally. Repeated administrations spaced over a 10 d interval produced glucose‐lowering results for a month with no hepatotoxicity detected. This study presents a platform on which nonviral oral delivery of nucleic acid therapeutics can be built.

## 5 Experimental Section

*Materials*: CS (*M*_W_ = 15 kDa) with ≈85% DA was purchased from Koyo Chemical Co. Ltd. (Hyogo, Japan), while succinic anhydride, bPEI (*M*_W_ = 0.8 and 25 kDa), EDC, and NHS were obtained from Sigma‐Aldrich (St Louis, MO, USA). The pEGFP‐N2 (4.7 kb) and INSL4 (5.3 kb) plasmids that were used in the investigation were purchased from Clontech (Palo Alto, CA, USA) and OriGene (Rockville, MD, USA), respectively. All other chemicals and reagents were of analytical grade.

*Plasmid Preparation*: The pEGFP‐N2 and INSL4 plasmids were separately amplified using DH5α and then purified using a Qiagen Plasmid Mega Kit (Valencia, CA, USA), following the manufacturer's instructions. The purity of the amplified plasmids was analyzed by gel electrophoresis (1.0% agarose), and their concentrations were measured using a NanoDrop 2000 Spectrophotometer (Thermo Fisher Scientific, Wilmington, DE, USA).

*Synthesis and Characterization of CS‐g‐bPEI*: A two‐step process for synthesizing CS‐g‐bPEI copolymers in a homogeneous aqueous environment was developed. In the first step, a predetermined amount of succinic anhydride (0.4, 1.2, 2.0, 2.8, or 3.6 g) was added to aqueous CS (1.0 g CS in 2% acetic acid, 80 mL). After it had been stirred at room temperature for 1 h, the mixture was adjusted to pH 7.0 and then precipitated by the addition of ethanol. The precipitate (succinated CS with various DSs) was filtered, washed with an excess of ethanol, and dried in a vacuum. In the second step, the succinated CS (0.1 g) and bPEI (0.8 kDa, 1 g) were dissolved in DI water (9 mL); the pH of the mixture was adjusted to 5.5 by adding 1 n HCl, and then EDC (0.29 g) and NHS (0.035 g) were added with magnetic stirring for 24 h to initiate the reaction. The synthesized CS‐g‐bPEI was dialyzed against DI water for 3 d to remove the unconjugated bPEI. The succinated CS and the CS‐g‐bPEI copolymer were characterized by ^1^H NMR (Avance 500, Bruker DRX, Rheinstetten, Germany) and FT‐IR (Perkin−Elmer Spectrum RX1 System, Buckinghamshire, UK).^16^

*Buffering Capacity of CS‐g‐bPEI*: The buffering capacity of the CS‐g‐bPEI copolymers was compared to those of unmodified CS and bPEI (25 kDa) by acid titration experiments.^18^ Test polymers were separately dissolved in 20 mL of 0.15 m NaCl solution to obtain the final concentration of 0.2 mg mL^−1^; the pH of each test solution was then adjusted to pH 10.0 using 0.1 n NaOH. The sample solutions were then titrated with 0.1 n HCl dropwise, and the pH of the solutions was monitored using a pH meter. An NaCl solution that contained no test polymers was the control.

*Preparation and Characterization of CS‐g‐bPEI/pDNA NPs*: The as‐synthesized CS‐g‐bPEI copolymer was dissolved in DI water to yield a 1% w/v stock solution, which was stored at 4 °C until used. Test NPs with various CP:pDNA weight ratios (ranging from 4:1 to 20:1) were obtained by adding 25 µL of aqueous CS‐g‐bPEI (40, 50, 60, 80, 100, or 200 µg), using a pipette, to an equal volume of aqueous pDNA (10 µg) and then thoroughly mixing the mixture for 30 s by vortexing, before leaving it for at least 1 h at room temperature. The size and zeta potential of the as‐prepared NPs were then obtained using a DLS (Zetasizer Nano‐ZS, Malvern, Worcestershire, UK) in DI water.

*Transfection Efficiency and Cytotoxicity of CS‐g‐bPEI/pDNA NPs*: GFP was used in the assessment of the in vitro transfection efficiency of CS‐g‐bPEI/pDNA NPs in the HT1080 cell line. Cells were seeded overnight on 24‐well plates at 8.0 × 10^4^ cells per well and then transfected at 70−90% confluency. Prior to transfection, the media were removed and replenished with 0.3 mL complete culture medium (DMEM supplemented with 10% FBS) that contained test NPs at a concentration of 1 µg pDNA per well. Twenty‐four hours following transfection, the cells were observed under a fluorescence microscope (Carl Zeiss Optical, Chester, VA, USA) or were harvested to evaluate their transfection efficiency by flow cytometry (Beckman Coulter, Fullerton, CA, USA).^20^ Their corresponding cytotoxicity was obtained by measuring the activity of LDH that was released from the cytosol of the treated cells using an LDH Cytotoxicity Assay Kit (Roche Diagnostics GmbH, Mannheim, Germany). Cells that were transfected with bPEI (25 kDa) were used as the positive control, and untreated cells were the untreated control.

*Physical Stability of CS‐g‐bPEI/pDNA NPs in Particular pH Environments*: To determine the physical stability of CS‐g‐bPEI/pDNA NPs during transit, their morphological changes at particular pH values that were used to simulate GI tract were examined by TEM (JEOL 2010F, Tokyo, Japan); their particle size and zeta potential were also determined using DLS.

*Protection of pDNA by CS‐g‐bPEI against Gastric pH and Intestinal Enzymes*: The ability of CS‐g‐bPEI to protect pDNA (1 µg) in the form of NPs under various pH conditions (pH 2.0, 6.0, 6.6, or 7.4) or against intestinal enzymes (first, DNAse I (4 µg mL^−1^, 25 µL) or DNAse I, followed by lysozyme (0.5 U mL^−1^, pH 5.5, 8 µL)) was evaluated.^20^ Enzymatic degradation was terminated by adding 5 µL of 0.5 m ethylene diaminetetraacetic acid (EDTA). The integrity of pDNA was evaluated by gel electrophoresis (1.0% agarose gel).

*Energy Dependence of Transcytosis of CS‐g‐bPEI/pDNA NPs*: The effects of temperature on the transepithelial transport of CS‐g‐bPEI/pDNA NPs were investigated in vitro using Caco‐2 cell monolayers that were grown on a tissue‐culture‐treated polycarbonate filter in a Costar Transwell with 12 wells per plate (Corning Costar Corp., Corning, NY, USA).^38^ Upon the addition of test NPs at 4 or 37 °C, the change in TEER, which began in the range of 600–800 Ω cm^2^, for the tightness of cell monolayers was measured using a Millicell‐Electrical Resistance System (Millipore Corp., Bedford, MA, USA).

*Transport of CS‐g‐bPEI/pDNA NPs Across Caco‐2 cell Monolayers*: In this study, Cy3‐labeled pDNA was synthesized using the Label IT Cy3 Nucleic Acid Labeling Kit (Mirus Corp., Madison, WI, USA); this Cy3‐pDNA was then used to prepare fluorescent NPs. The fluorescent NPs (5 µg pDNA per well) were introduced into the donor compartment of the Caco‐2 cell monolayers. Following incubation for 3 h at 4 or 37 °C, test NPs were aspirated. After fixation, the *Z*‐stack images of the cell monolayers were obtained using an inverted CLSM (Zeiss LSM780, Carl Zeiss, Jena GmbH, Jena, Germany). Meanwhile, samples were extracted from the receiving compartment; the intensity of the fluorescent NPs was evaluated using a microplate spectrophotometer (SpectraMax M5, Molecular Devices, Sunnyvale, CA, USA), and the *P*_app_ value (cm s^−1^) of the NPs was calculated as described elsewhere.^22^

*Animal Study*: ICR mice (around 30 g, BioLasco, Ilan, Taiwan) were used in the animal experiments, which were performed in compliance with the “Guide for the Care and Use of Laboratory Animals,” which was published by the National Academy Press in 1996. The Institutional Animal Care and Use Committee of National Tsing Hua University approved all studies.

*Biodistribution of CS‐g‐bPEI/pDNA NPs and Their Gene Transfection*: In the biodistribution study, Cy5‐labeled CS (Cy5‐CS) was synthesized using Cy5‐NHS ester according to the manufacturer's protocol (Lumiprobe Corp., Broward, FL, USA); the synthesized Cy5‐CS was used to prepare fluorescent NPs. To prevent autofluorescence, the test mice were fed with a special alfalfa‐free diet for at least 3 d before and during the experiments. Mice that had fasted overnight were restrained by hand and orally administered fluorescent NPs that contained 1 mg pEGFP‐N2 suspension (200 µL), using an oral feeding needle. At predetermined intervals (12 or 48 h) after treatment, the test mice were sacrificed, and their organs were harvested and visualized by IVIS (Xenogen, Alameda, CA, USA) to observe the biodistribution of fluorescent NPs or their expression of GFP (*n* = 3 in each group).^39^

*Immunohistochemistry for Detection of Kupffer Cells and Human Insulin*: To detect Kupffer cells in liver tissues, cryosections were fixed and then blocked with 5% goat serum at 37 °C for 1 h. After they had been washed in phosphate‐buffered saline (PBS), the sections were incubated with an antimouse F4/80 antibody (eBioscience, San Diego, CA, USA) at 1:100 dilution, followed by a Cy3‐conjugated goat antimouse immunoglobulin G (IgG) (Invitrogen, Carlsbad, CA, USA) at 1:50 dilution. Sections were counterstained with 4′,6‐diamidino‐2‐phenylindole (DAPI) to visualize the nuclei. To detect human insulin, cryosections were fixed and then blocked with Vector MOM kit (Vector Laboratories Inc., Burlingame, CA, USA). Section slides were then stained with a mouse anti‐DDK monoclonal antibody (Origene, Rockville, MD, USA) at 1:100 dilution, before being treated with Dylight 633‐conjugated goat antimouse IgG (Invitrogen, Carlsbad, CA, USA) at 1:50 dilution and subsequently counterstained with DAPI.

*Glucose‐Lowering Effects*: Diabetes was induced in test mice by the intraperitoneal injection of STZ (50 mg kg^−1^ d^−1^ for 4 d). Mice with fasting blood glucose levels above 300 mg dL^−1^ were regarded as diabetic and included in the study.^40^ The following formulations were, individually, orally administered to the diabetic mice: DI water (untreated control), naked insulin plasmid (600 µg pDNA in 200 µL DI water), and insulin plasmid NPs (containing 600 µg pDNA in 200 µL DI water), once every 10 d for a total of 30 d (*n* = 6 for each group). Blood samples were collected from the tail vein, and the fasting blood glucose levels were determined using a hand‐held glucose meter (LifeScan Inc., Milpitas, CA, USA) at 4 h after fasting. The plasma insulin levels were also analyzed using an Ultrasensitive Insulin ELISA kit (Mercodia, Uppsala, Sweden).

*Enhancement of ^18^F‐FDG Utilization*: Glucose utilization was evaluated by administering ^18^F‐FDG (11.1 MBq in 0.2 mL) to the same diabetic mice on day 2 following the oral ingestion of DI water or insulin plasmid‐loaded NPs (600 µg pDNA). Dynamic PET images were obtained using a PET scanner at 1 h after treatment (Siemens Medical Solutions, Knoxville, TN, USA). These images were reconstructed using the 2D ordered subset expectation maximization (OSEM) method (4 iterations and 16 subsets) with attenuation corrections. After PET scanning, whole‐body CT images were obtained using NanoSPECT/CT (Bioscan, Washington, DC, USA).^28^ 3D image, ^18^F‐FDG PET, and CT scans were fused using the image workstation and PMOD version 3.7 (PMOD Technologies Ltd., Zurich, Switzerland). Regions of interest (ROI) were manually drawn over the following tissues: neck muscle, scapular muscle, masseter muscle, front leg muscle, and hind leg muscle. Tracer uptake by various tissues was quantified as mean percentage injected dose per gram (%ID g^−1^), calculated as ROI activity divided by injected dose multiplied by 100%.

*Acute Liver Toxicity*: The acute liver toxicities of the animals that had been treated with DI water (untreated control), naked insulin plasmid, or insulin plasmid NPs were obtained by measuring serum ALT and AST activities over time.^41^ Biopsy specimens of the liver were fixed in 10% phosphate‐buffered formalin, embedded in paraffin, sectioned with a thickness of 5 µm, and then stained with hematoxylin and eosin (H&E).

*Statistical Analysis*: All results were presented as mean ± standard deviation. To compare the means between pairs of groups, the two‐tailed Student‐*t* test was used. Differences were considered to be statistically significant at *P* < 0.05.

## Conflict of Interest

The authors declare no conflict of interest.

## Supporting information

Supplementary
